# Supplementary material for: Commercial Poultry Production Stocking Density Influence on Bird Health and Performance Indicators
Source: Animals (Basel). 2020 Jul 23;10(8):1253. doi: 10.3390/ani10081253 (PMC7460447; doi:10.3390/ani10081253)
Supplement: Supplementary file 1 [file animals-10-01253-s001.pdf]

animals

Supplementary Files: Commercial Poultry Production Stocking Density Influence on Bird Health and Performance Indicators

Stéphane Bergeron [1,*], Emmanuelle Pouliot [2] and Maurice Doyon [2]

Table S1. Regression models with statistics including standard error (Std. Error), and t-value.

Male - ADG model	Coefficients	Std. Error	t value	Pr(>|t|)	
Intercept	4.03E+01	1.06E+01	3.811	0.00025	***
Age	2.55E-01	3.25E-01	0.786	0.43382	
Area	9.45E-05	7.03E-04	0.134	0.89345	
Density	5.45E-01	1.05E-01	5.171	1.35E-06	***
2nd trimester	7.59E-01	1.28E+00	0.595	0.55302	
3rd trimester	2.09E+00	1.09E+00	1.909	0.05938	,
4th trimester	1.05E+00	1.05E+00	1.004	0.3179	

Residual standard error: 3,78 on 92 degrees of freedom
Multiple R-squared: 0,3545, Adjusted R-squared: 0,3124
F-statistic: 8,421 on 6 and 92 DF, p-value: 2,757e-07

Signif, codes: 0 '***' 0,001 '**' 0,01 '*' 0,05 ',' 0,1 ' ' 1

Male - Condemnation model	Coefficients	Std. Error	t value	Pr(>|t|)
Intercept	-3.38E-03	4.69E-02	-0.072	0.943
Age	-4.40E-05	1.44E-03	-0.031	0.976
Area	-2.18E-07	3.12E-06	-0.07	0.945
Density	6.61E-04	4.67E-04	1.414	0.161
2nd trimester	3.06E-04	5.65E-03	0.054	0.957
3rd trimester	6.83E-03	4.85E-03	1.408	0.163
4th trimester	7.42E-03	4.64E-03	1.598	0.113

Residual standard error: 0,01676 on 92 degrees of freedom
Multiple R-squared: 0,08692, Adjusted R-squared: 0,02738
F-statistic: 1,46 on 6 and 92 DF, p-value: 0,2008

Signif, codes: 0 '***' 0,001 '**' 0,01 '*' 0,05 ',' 0,1 ' ' 1

Male - %Grade A meat					
	Coefficients	Std. Error	t value	Pr(>|t|)	
Intercept	84.678609	7.3145272	11.577	<2e-16	***
Age	0.2087692	0.2245294	0.93	0.355	
Area	-0.0002961	0.0004865	-0.609	0.544	
Density	0.0424013	0.0728726	0.582	0.562	
2nd trimester	1.9955588	0.8816734	2.263	0.026	*
3rd trimester	0.8332339	0.7566384	1.101	0.274	
4th trimester	0.9716737	0.7240471	1.342	0.183	

Residual standard error: 2,615 on 92 degrees of freedom

Multiple R-squared: 0,07232, Adjusted R-squared: 0,01182

F-statistic: 1,195 on 6 and 92 DF, p-value: 0,3159

Signif, codes: 0 '***' 0,001 '**' 0,01 '*' 0,05 ',' 0,1 ' ' 1

Male - %Pod-0					
	Coefficients	Std. Error	t value	Pr(>|t|)	
Intercept	5.69E-01	7.53E-01	0.755	0.452	
Age	7.29E-03	2.31E-02	0.316	0.753	
Area	-3.02E-05	5.01E-05	-0.602	0.5485	
Density	-1.44E-03	7.50E-03	-0.192	0.8484	
2nd trimester	-1.95E-01	9.08E-02	-2.151	0.0341	*
3rd trimester	-1.55E-01	7.79E-02	-1.986	0.0501	
4th trimester	1.74E-02	7.45E-02	0.234	0.8158	

Residual standard error: 0,2691 on 92 degrees of freedom

Multiple R-squared: 0,1162, Adjusted R-squared: 0,05852

F-statistic: 2,015 on 6 and 92 DF, p-value: 0,07143

Signif, codes: 0 '***' 0,001 '**' 0,01 '*' 0,05 ',' 0,1 ' ' 1

Male - Mortality Rate					
	Coefficients	Std. Error	t value	Pr(>|t|)	
Intercept	-5.02E-02	5.63E-02	-0.89	0.3756	
Age	3.34E-03	1.73E-03	1.931	0.0566	.
Area	3.25E-06	3.75E-06	0.867	0.3883	
Density	-1.09E-03	5.61E-04	-1.934	0.0562	.
2nd trimester	7.53E-04	6.79E-03	0.111	0.912	
3rd trimester	2.65E-03	5.83E-03	0.454	0.6508	

4th trimester	1.05E-02	5.58E-03	1.881	0.0631	.

Residual standard error: 0.02014 on 92 degrees of freedom

Multiple R-squared: 0.1661, Adjusted R-squared: 0.1117

F-statistic: 3.055 on 6 and 92 DF, p-value: 0.009032

Signif. codes: 0 '***' 0.001 '**' 0.01 '*' 0.05 '.' 0.1 ' ' 1

Female - ADG Model					
	Coefficients	Std. Error	t value	Pr(>|t|)	
Intercept	39.2612451	6.7565941	5.811	7.58E-08	***
Age	0.2163757	0.1800378	1.202	0.23229	
Area	0.0004252	0.0003923	1.084	0.28106	
Density	0.386999	0.0763183	5.071	1.85E-06	***
2nd trimester	-0.0208544	0.6509213	-0.032	0.97451	
3rd trimester	1.867041	0.6550531	2.85	0.00532	**
4th trimester	1.1937982	0.6734822	1.773	0.07938	,

Residual standard error: 2.338 on 99 degrees of freedom

Multiple R-squared: 0.3903, Adjusted R-squared: 0.3533

F-statistic: 10.56 on 6 and 99 DF, p-value: 4.88e-09

Signif. codes: 0 '***' 0.001 '**' 0.01 '*' 0.05 '.' 0.1 ' ' 1

Female - Condemnations					
	Coefficients	Std. Error	t value	Pr(>|t|)	
	Estimate	Std. Error	t value	Pr(>|t|)	
Intercept	3.79E-03	1.58E-02	0.24	0.811078	
Age	1.65E-04	4.21E-04	0.392	0.696258	
Area	-3.85E-07	9.17E-07	-0.419	0.675874	
Density	1.26E-04	1.79E-04	0.706	0.481543	
2nd trimester	-2.87E-03	1.52E-03	-1.886	0.062178	,
3rd trimester	-2.73E-03	1.53E-03	-1.782	0.07777	,
4th trimester	5.62E-03	1.58E-03	3.569	0.000555	***

Residual standard error: 0.005466 on 99 degrees of freedom

Multiple R-squared: 0.3393, Adjusted R-squared: 0.2993

F-statistic: 8.473 on 6 and 99 DF, p-value: 1.991e-07

Signif. codes: 0 '***' 0.001 '**' 0.01 '*' 0.05 '.' 0.1 ' ' 1

Female - %Grade A meat				
	Coefficients	Std. Error	t value	Pr(>|t|)

Intercept	1.01E+02	8.13E+00	12.465	<2e-16	***
Age	-9.74E-02	2.17E-01	-0.45	0.654	
Area	-5.60E-04	4.72E-04	-1.187	0.238	
Density	-1.25E-01	9.18E-02	-1.358	0.177	
2nd trimester	1.15E-01	7.83E-01	0.146	0.884	
3rd trimester	7.48E-01	7.88E-01	0.95	0.345	
4th trimester	5.35E-01	8.10E-01	0.661	0.51	

Residual standard error: 2.811 on 99 degrees of freedom

Multiple R-squared: 0.03711, Adjusted R-squared: -0.02125

F-statistic: 0.6359 on 6 and 99 DF, p-value: 0.7012

Signif. codes: 0 '***' 0.001 '**' 0.01 '*' 0.05 '.' 0.1 ' ' 1

Female - %Podo-0

	Coefficients	Std. Error	t value	Pr(>|t|)	
Intercept	152.69299	72.712475	2.1	0.0383	*
Age	-1.178394	1.937514	-0.608	0.5444	
Area	-0.007028	0.004222	-1.665	0.0991	,
Density	-0.417994	0.821315	-0.509	0.6119	
2nd trimester	-16.359049	7.005023	-2.335	0.0215	*
3rd trimester	-7.351737	7.049489	-1.043	0.2995	
4th trimester	-2.787208	7.247817	-0.385	0.7014	

Residual standard error: 25.16 on 99 degrees of freedom

Multiple R-squared: 0.0859, Adjusted R-squared: 0.0305

F-statistic: 1.55 on 6 and 99 DF, p-value: 0.1698

Signif. codes: 0 '***' 0.001 '**' 0.01 '*' 0.05 '.' 0.1 ' ' 1

Female - Mortality Rate

	Coefficients	Std. Error	t value	Pr(>|t|)	
Intercept	-5.60E-02	6.11E-02	-0.916	0.36188	
Age	4.18E-03	1.63E-03	2.57	0.01165	*
Area	-4.34E-06	3.55E-06	-1.224	0.22399	
Density	-2.18E-03	6.90E-04	-3.163	0.00207	**
2nd trimester	1.05E-02	5.89E-03	1.775	0.07896	.
3rd trimester	2.01E-03	5.92E-03	0.339	0.73504	
4th trimester	6.78E-03	6.09E-03	1.114	0.26798	

Residual standard error: 0.02114 on 99 degrees of freedom

Multiple R-squared: 0.1807, Adjusted R-squared: 0.1311

F-statistic: 3.64 on 6 and 99 DF, p-value: 0.00263	
Signif. codes: 0 '***' 0.001 '**' 0.01 '*' 0.05 '.' 0.1 ' ' 1	
